# Supplementary material for: MSA-VT Score for Assessment of Long-Term Prognosis after Electrical Storm Ablation
Source: Biomedicines. 2024 Feb 22;12(3):493. doi: 10.3390/biomedicines12030493 (PMC10968369; doi:10.3390/biomedicines12030493)
Supplement: Supplementary file 1 [file biomedicines-12-00493-s001.zip › biomedicines-2848461-supplementary.pdf]

Supplementary materials

Manuscript title:" MSA-VT score for assessment of long-term prognosis after electrical storm ablation"

Journal: Biomedicines

Supplementary Table S1

	Death during follow-up, % (n)	p
Sex		
Males	42.9% (n = 25)	0.35
Females	28.7% (n = 6)	
BMI ≥ 25 kg/sqm		
Yes	28.6% (n = 10)	0.82
No	31.8% (n = 21)	
COPD		
Yes		
No		
T2DM		
Yes	38.5% (n = 10)	0.33
No	28% (n = 21)	
Hypertension		
Yes	34.9% (n = 22)	0.27
No	23.7% (n = 9)	
Dyslipidemia		
Yes	32.3% (n = 20)	0.82
No	28.2% (n = 11)	
Smoker		
Yes	21.1% (n = 4)	0.41
No	32.9% (n = 27)	
CKD		
Yes	31.6% (n = 6)	0.99
No	29.6% (n = 24)	
Ischemic cardiomyopathy		
Yes	27.9% (n = 19)	0.49
No	36.4% (n = 12)	
Previous CRT, % (n)		
Yes	33.3% (n = 5)	0.77
No	30.2% (n = 26)	
BB prior to ES		
Yes	31.3% (n = 26)	0.99
No	27.8% (n = 5)	
BB after ES ablation		
Yes	31% (n = 27)	0.99
No	28.6% (n = 4)	
Amiodarone prior to ES		
Yes	35.3% (n = 24)	0.17
No	21.2% (n = 7)	
Amiodarone after ES ablation		
Yes	34.7% (n = 25)	0.24
No	20.6% (n = 6)	
**AF at admission, % (n)**		

Yes	69.2% (n = 9)	**0.003**
No	25% (n = 22)	
**History of AF, % (n)**		
Yes	47.5% (n = 19)	**0.004**
No	19.7% (n = 12)	
**Moderate/severe MR, % (n)**		
Yes	54.5% (n = 18)	**< 0.001**
No	19.1% (n = 13)	
**NYHA III-IV at admission, % (n)**		
Yes	51.7% (n = 15)	**0.008**
No	22.2% (n = 16)	
**Residual SMVT**		
Yes	59.4% (19)	**< 0.001**
No	17.4% (12)	
Previous ablation		
Yes	28.1% (n = 9)	0.81
No	31.9% (n = 22)	
**Recurrence**		
Yes	55.6% (n = 20)	**< 0.001**
No	16.9% (n =11)	
Acute ablation		
Yes	60% (n = 3)	0.16
No	29.2% (n = 28)	

Supplementary Table S1. Rates of death during follow-up in relation to categorical parameters regarding clinical and procedural characteristics. SMVT = sustained monomorphic ventricular tachycardia, NYHA = New York Heart Association, MR = mitral regurgitation, AF = atrial fibrillation, ES = electrical storm, BB = beta blocker, CKD = chronic kidney disease, T2DM = type 2 diabetes mellitus, BMI = body-mass index, CRT = cardiac resynchronization therapy

Supplementary Table S2

	Alive	Dead	p
**Age (years)**	**57 ± 13.2**	**65.5 ± 9.7**	**0.002**
**Baseline creatinine (mg/dl)**	**1 ± 0.3**	**1.3 ± 0.9**	**0.03**
**LVEF (%)**	**33.4 ± 12.4**	**28.7 ± 9**	**0.03**
End-diastolic LV diameter (mm)	59.7 ± 10.1	62.4 ± 11.8	0.42
Number of procedures	1.3 ± 0.5	1.4 ± 0.7	0.95
**Procedure length (min)**	**188.9 ± 77.5**	**235.1 ± 96.2**	**0.02**
Days of hospitalization (days)	9.2 ± 9	11.6 ± 11.4	0.26
**PAINESD score (points)**	**13.6 ± 6**	**16.5 ± 5.9**	**0.028**
**RIVA score (points)**	**11.3 ± 4.4**	**14.5 ± 4.7**	**0.002**
**I-VT score (mean HR)**	**0.8 ± 1.2**	**1.75 ± 2.1**	**0.02**

Supplementary Table S2. Mean values of numerical variables in relation to mortality during follow-up. LVEF = left ventricular ejection fraction, LV = left ventricle

Supplementary Figure S1

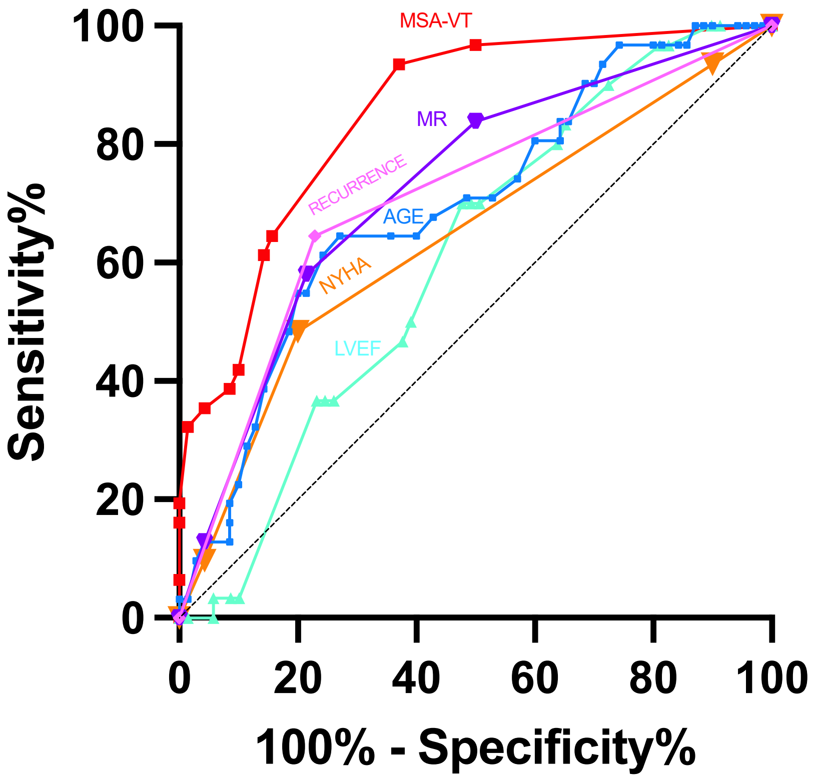

	AUC	p
MR	0.72	< 0.001
AGE	0.70	< 0.001
NYHA	0.65	0.01
LVEF	0.39	0.09
MSA-VT	0.84	< 0.001
RECURRENCE	0.71	< 0.001

Supplementary Figure S1. Receiver operator curve analysis regarding the MSA-VT score compared to singular risk factors for all-cause mortality during follow up. LVEF = left ventricular ejection fraction, NYHA = New York Heart Association, MR = moderate or severe mitral regurgitation

Supplementary Figure S2

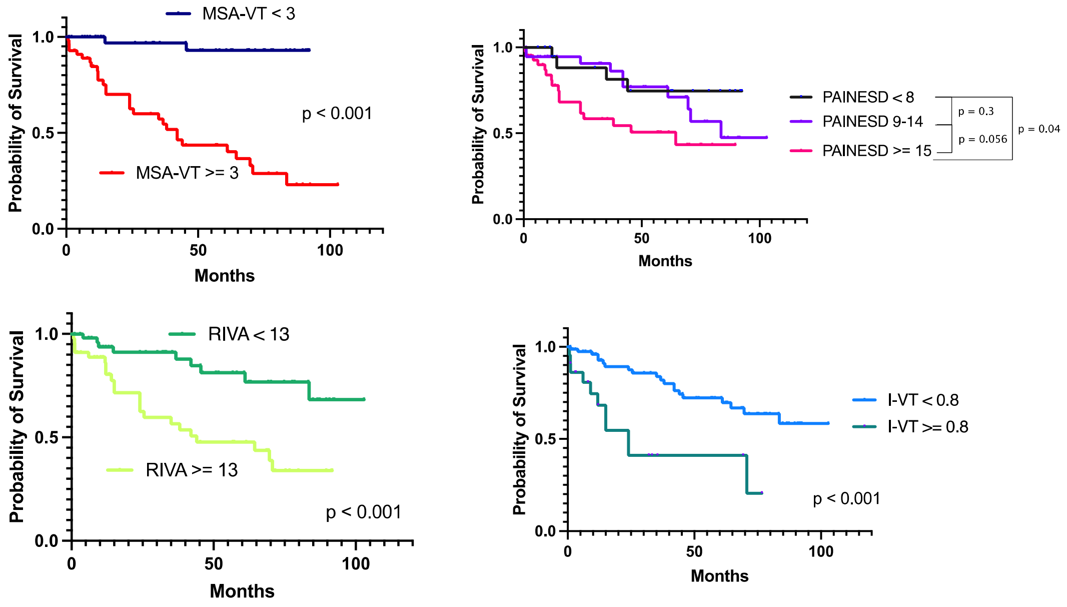

Supplementary Figure S2. Survival curves plotted by Kaplan-Meier method and compared by pairwise log-rank test. Based on log-rank test MSA-VT score (cut-off 3 points), RIVA (cut-off 13 points), I-VT (cut-off 0.8 points in simplified classification low versus non-low risk as stratified by authors) patients with scores higher than established threshold show significantly higher all-cause mortality during long-term follow-up; PAINESD scoring algorithm shows that the only patients with PAINESD scores < 8 have significantly lower all-cause mortality during long-term follow compared to those with PAINESD scores ≥ 15, whereas the other subgroups do not demonstrate significantly different all-cause mortality at pairwise log-rank comparison
